# Is legislation to prevent genetic discrimination necessary in Japan? An overview of the current policies and public attitudes

**DOI:** 10.1038/s10038-023-01163-z

**Published:** 2023-06-08

**Authors:** Kaori Muto, Akiko Nagai, Izen Ri, Kyoko Takashima, Sachie Yoshida

**Affiliations:** 1 Department of Public Policy, The Institute of Medical Science, The University of Tokyo, Minato-ku, Tokyo, Japan; 2 Bioethics Section, Center for Clinical Sciences, National Center for Global Health and Medicine, Shinjuku-ku, Tokyo, Japan; 3 Department of Biomedical Ethics, Kyoto Prefectural University of Medicine, Kyoto City, Kyoto Japan

**Keywords:** Ethics, Personalized medicine

## Abstract

Genetic discrimination (GD) has not been discussed in East Asia as extensively as in Europe and North America. Influenced by UNESCO’s universal declaration in 1997, the Japanese government took a stringent approach toward GD by releasing the Basic Principles on Human Genome Research in 2000. However, Japanese society has mostly been ignoring the prevention of GD for decades, and the principle of prohibiting GD was never adhered to in any of the Japanese laws. We conducted anonymous surveys among the general adult population in 2017 and 2022 to explore their experiences of GD and attitudes toward laws carrying penalties to prevent GD in Japan. In both years, approximately 3% of the respondents had experienced some unfavorable treatment regarding their genetic information. They showed higher recognition of the benefits of using genetic information and lower recognition of concerns about using genetic information and GD in 2022 than in 2017. However, the awareness regarding the need for legislation with penalties on GD had increased over the five-year period. In 2022, the framework of a bill to promote genomic medicine and prevent GD without any relevant penalties was released by the Bipartisan Diet Members Caucus. Considering that the absence of regulations may be a barrier to obtaining genomic medicine, as the initial step toward making the prohibition of GD more effective, legislation that no form of GD will be tolerated may stimulate education and awareness regarding respect for the human genome and its diversity.

## Introduction

Genetic discrimination (GD) is a classic example of ethical, legal, and social implications/issues in genetic research and medicine. UNESCO’s 1997 Universal Declaration on the Human Genome and Human Rights states that “no one shall be subjected to discrimination based on genetic characteristics that is intended to infringe or has the effect of infringing human rights, fundamental freedoms and human dignity [1].” Multiple definitions of GD exist, but a common feature is the actual or perceived unfair or negative treatment of an individual or a group based on genetic differences [2, 3]. Compared to North America and Europe, where legislation, moratoria, or administrative regulations regarding the use of genetic test results have been adopted, GD has generated less discussion in East Asia. Aside from South Korea, none of the seven Asian jurisdictions has adopted specific legislation to prevent GD [4]. In particular, Japan has not adopted specific laws, regulations, or policies to prevent GD [5]. For decades, the Japanese government, the Diet of Japan, and academia did not take any concrete measures for the prevention of GD; however, in 2022, the debate in the Diet regarding this finally started. The purpose of this article is to provide an overview of the policies against GD in Japan to date, share the results of surveys regarding people’s attitudes toward GD, and suggest some actions that should be taken.

## History of Japanese policies against genetic discrimination

Influenced by UNESCO’s Declaration in 1997—which was one year after the Eugenic Protection Law of Japan was finally repealed—the Japanese government took a tough stance against GD by releasing the Basic Principles on Human Genome Research in 2000 [6]. Article 16 states that “donors shall not be discriminated against on the basis of genetic characteristics indicated by their own genetic information revealed as a result of research,” “there can be a variety of specific types of discrimination, such as employment, insurance, and marriage,” and “efforts should be made to prohibit or eliminate discriminatory treatment within the framework of existing laws and systems, and appropriate institutional measures should be taken in the future, including the possibility of enactment of new laws and regulations.” These principles harmonize with UNESCO’s Declaration and should have been respected.

However, the principal nuance of the prohibition of GD disappeared from the Ethical Guidelines for Human Genome/Gene Analysis Research, which were developed jointly by the Ministry of Education, Culture, Sports, Science and Technology (MEXT), the Ministry of Health, Labour and Welfare (MHLW), and the Ministry of Economy, Trade and Industry (METI) in 2001 [7]. The guidelines stated that (1) researchers were responsible for explaining “the foreseeable risks and disadvantages (including disadvantages in social life such as social discrimination)” to research participants and (2) the principal investigator should report to the head of their research institution when discrimination may occur as a result of the use of minors’ genetic information. The guidelines did not require that researchers make any effort to prevent GD. Furthermore, with the repeal and alteration of the new ethical guidelines in 2021, these descriptions were removed in their entirety from the main text [8].

With regard to guidelines on genetic medicine, 10 Japanese academic societies related to genetic medicine jointly issued a set of guidelines in 2003, which included the following: (1) even if the person tested provides consent, the test results must not be accessible to employers, insurance companies, or schools; (2) healthcare professionals engaged in genetic testing must always exercise caution and special care to ensure that genetic test results are not used in any discriminatory way; and (3) genetic test results must not be used to discriminate against a person who has been tested. As the people tested for the purpose of predicting the onset of a particular disease are generally healthy, strict protections of privacy and appropriate psychological support must be provided [9]. In particular, requirements were imposed on healthcare providers to ensure that people who were tested were not discriminated against in the context of education, employment (including promotions), and insurance coverage. While all of these statements were removed when the guidelines were revised in 2011 [10], some of them were restored in the 2022 revision [11].

## Can GD be prevented under the current legislation in Japan?

The Personal Data Protection Law was amended in 2015, and the terms “individual identification code” and “special-care-required personal information” were added. The description “base sequence constituting DNA taken from a cell” was included into the category of “individual identification code.” However, it was still unclear how these statutory definitions, especially “special-care-required personal information”, should be interpreted and operationalized in genomic medicine. The MHLW set up a task force to discuss how genetic test results, sequenced data, and relevant information fit into the new legal terms [12]. Consequently, “genetic information” was defined as information transmitted to progeny such as genetic test results to detect germline variants. In addition, “genomic information” was defined as information containing interpretations of certain sequence data including both somatic and germline variants. It was decided that both pieces of information would be included in “special-care-required personal information” in the law.

The report of the task force also acknowledged that “there are no legal regulations that directly prohibit discrimination based on genetic characteristics,” but the suggested response was vaguely expressed as “it is necessary to promote efforts to develop the social environment necessary for the promotion of genomic medicine.” Although several members of the task force mentioned the need for legislation on several occasions during the nine meetings [12], the MHLW, which compiled the report, did not acknowledge the need for legislation and limited itself to explaining what could be done within the framework of existing laws and regulations.

For example, the MHLW’s recommendations to employers encouraged them to use only the aptitude and abilities necessary to perform a job as the criteria for employment selection. In the area of employment management, the MHLW has stipulated since 2004 that health information should not be handled beyond the scope necessary to ensure employees’ health. The Insurance Business Law stipulates that insurance companies must obtain approval from the Financial Services Agency (FSA) regarding their business methods, general insurance policy conditions, and methods of calculating premiums and policy reserves when selling new life insurance products. The FSA is responsible for approving insurance contracts to prevent unfair discrimination against people.

The report of the MHLW task force concluded [13], “it is necessary to recognize that there are issues to be addressed in legally stipulating GD, such as the need to clarify the acts to be covered by the law and to ensure consistency between the handling of genomic information and non-genomic information.” The task force also advocated that the public be surveyed regarding their concerns “about the provision of genomic information.”

In the absence of a legal ban against GD, approximately 100 genetic tests are covered by universal health insurance in Japan. One such test, a tumor-profiling gene panel test, has been performed about 45,000 times since its inclusion in universal health insurance coverage in 2019.

## New bill to promote genomic medicine and prevent GD

Although a new policy group on genomic medicine comprising bipartisan parliamentarians was formed in 2015, it took some time for them to decide whether to create any new legislation. While various needs were eagerly expressed by the long-neglected genomic medicine community, the lawmakers did not evince much interest in improving genomic medicine and prohibiting GD. However, the MHLW began its policy of promoting genomic medicine in earnest in 2017 without the government and the Diet taking any precautionary measures for GD, which resulted in great concerns among the patient community.

With the support of Genetic Alliance in the US as a coalition of patient associations for hereditary diseases, Genetic Alliance Japan was established in 2017. The organization, headed by Ms. Makiko Dazai, submitted requests for a unified response to GD by relevant government ministries, academia, and industry, in addition to the establishment of legislation to prevent GD. The Japan Federation of Cancer Patient Groups, an umbrella organization of cancer patient associations headed by Mr. Shinsuke Amano, also submitted a request for legislation to the ruling parties in 2018 and held a rally at the Diet Members’ Building in December 2019 for the promotion of cancer genome medicine and the protection of patients and others from social disadvantages.

In 2021, the Bipartisan Diet Members Caucus released the framework of a bill, the basic principles of which were the promotion of genomic medicine, the consideration of bioethics, and the prevention of GD. However, the group was unable to reach a consensus within the ruling Liberal Democratic Party, and the bill was not drafted.

In April 2022, the presidents of the Federation of Medical Societies of Japan and the Japan Medical Association issued a joint statement urging the government and the Diet to regulate GD [14]. In May 2022, separate statements of the same nature directed at healthcare professionals on the current handling of genetic information were released by the Life Insurance Association of Japan and the General Insurance Association of Japan. The statement said, “we do not collect or use the results of genetic tests” even “where the results of genetic tests are included in the submitted notification or medical certificate.” It also declared clearly “if the name of the disease, family medical history, or record of genetic counseling by a physician is included in the submitted notification or medical certificate, we will not collect or use the results [15, 16].”

In October 2022, the final framework of the bill was released by the Bipartisan Diet Members Caucus. Patient advocacy groups, industry, and academic societies jointly lobbied for the early passage of the bill, with approximately 250 groups supporting its framework. These requests led to the swift drafting of the bill, and the final version was released in December.

The bill includes three basic principles. The first principle is to realize “world-class genomic medicine” in a wide range of medical fields and to make its benefits widely available to the public by promoting measures for research, development, and provision of genomic medicine in an organic, mutually coordinated manner. The second principle is “to ensure that appropriate consideration is given to bioethics at each stage of research, development, and provision of genomic medicine, given that some of these activities involve the manipulation of genes that can be passed on to progeny, which may have a significant impact on the preservation of human dignity”. This principle implies a cautious attitude toward germline editing in assisted reproductive technology; however, it fails to prohibit any activity. The third principle is that “genomic information obtained in the research and development and provision of genomic medicine should be sufficiently protected, and unjust discrimination should not be made based on the genomic information”. The bill requires the national and local governments to formulate a basic plan for “genomic medicine policies” based on these principles and does not define the relevant penalties.

As of this writing (June 5, 2023), this bill has been passed by the House of Representatives and is awaiting debate in the House of Councillors. If this bill is enacted, it will be the legal responsibility of the Japanese government to create measures to address GD.

## Public attitudes toward the regulation to prevent GD in Japan

More than 25 years have passed between the UNESCO Declaration and the completion of the bill containing the article preventing GD in Japan. Aside from the activities of patient communities, people’s views and experiences with GD remain unknown. We therefore conducted large-scale surveys to clarify people’s experiences of GD in Japan, their expectations and concerns about the use of genetic information, and their attitudes toward legal regulations to prevent GD and the inappropriate use of genetic information.

The situation surrounding genomic medicine has changed since 2019, and the people of Japan are more familiar now with genomic medicine. As such, we also conducted comparisons between the survey results in 2017 and 2022.

In February 2017 and April 2022, we distributed anonymous, cross-sectional online surveys to 44,360 and 45,488 adults aged 20–69 years from the general Japanese population. These samples were extracted based on national census data from the survey panel of INTAGE Inc.

The survey questions covered three topics: genetics knowledge, attitudes toward the use of genetic information, and the need for regulations to prevent GD and the inappropriate use of genetic information. The inclusion of family medical history in the definitions of GD prevention laws varies from country to country, and it is not clear whether genetic information includes family medical history in Japan. To ascertain the impact of genetic information in the broadest sense, the surveys were administered by explaining to the respondents that family history was included in genetic information.

We compared the characteristics and attitudes of the respondents toward the use of genetic information in 2017 and 2022 using chi-square tests and Wilcoxon rank-sum tests. Multivariate logistic regression analyses were performed using SAS version 9.4 (SAS Institute Inc., Cary, NC) to identify the factors associated with their attitudes toward legal regulations.

The response rates in 2017 and 2022 were 24.5% (10,881/44,360) and 11.6% (5268/45,488), respectively. We excluded 286 respondents who did not answer the questions on educational background in 2022 (*n* = 4982).

Table 1 shows the respondents’ characteristics. No significant gender differences were evident between 2017 and 2022, and the respondents’ mean ages were 46.1 and 46.2 years, respectively. In both surveys, approximately 3% of the respondents said they had experienced some form of GD, with some having experienced it in marriage, pregnancy, or childbirth. The percentage of respondents willing to undergo genetic testing at medical institutions was 27.9% and 21.7% in 2017 and 2022, respectively.

**Table 1:** Respondents’ characteristics

	2017		2022		
	*N*	%	*N*	%	*P*-value^*1^
Total	10,881		4982		
Gender					0.624
Male	5397	49.6	2492	50.0	
Female	5484	50.4	2490	50.0	
Age group (years)					<0.0001
20–29	1666	15.3	769	15.4	
30–39	2091	19.2	875	17.6	
40–49	2591	23.8	1160	23.3	
50–59	2101	19.3	1154	23.2	
60–69	2432	22.4	1024	20.6	
Marital status					<0.0001
Unmarried	3244	29.8	1707	34.3	
Married	7637	70.2	3275	65.7	
Do you have any children?					<0.0001
No	4904	45.1	2459	49.4	
Yes	5977	54.9	2523	50.6	
Educational background					<0.0001
Junior high school or high school	3697	34.0	1700	34.1	
Occupational school or junior college	2689	24.7	1416	28.4	
University or graduate school	4495	41.3	1866	37.5	
Have you undergone genetic testing in medical institutions?					
Yes	210	1.9	91	1.8	0.658
No or “Cannot remember”	10,671	98.1	4891	98.2	
Have you or your family members ever received unfavorable treatment regarding genetic information?^*2^					
Experienced some form of unfavorable treatment	351	3.2	163	3.3	0.189
Had no experiences of the above or did not wish to answer	10,530	96.8	4819	96.7	
Do you have willingness to undergo genetic testing at medical institutions?					
Yes	3038	27.9	1083	21.7	<0.0001
No or “Cannot decide”	7843	72.1	3899	78.3	

^*1^Chi-square tests were performed to assess the differences between respondents in 2017 and 2022

^*2^The respondents were required to answer multiple-choice questions about their experiences of unfavorable treatment based on genetic information regarding purchasing insurance, employment, relationships, marriage, pregnancy or childbirth, bullying at school or office, being refused participation in community events, or other treatment

Those who believed genetic information would be useful for disease prevention (the sum of “agree” and “tend to agree”) comprised 65.4% of the respondents in 2017 and 68.9% in 2022 (Table 2A). Nevertheless, 47.0% (2017) and 40.9% (2022) were concerned about the handling of genetic information in administrative agencies, 43.8% and 40.3% about GD in insurance, 37.6% and 33.8% about GD at work, and 41.0% and 35.6% about GD in marriage and pregnancy, respectively. The respondents in 2022 perceived genetic information to have more benefits (*p* < 0.0001) and had less concerns about genetic information than those in 2017 (*p* < 0.0001).

**Table 2:** A. Attitudes toward the benefits of and concerns about use of genetic information and genetic discrimination (GD) (%). B. Attitudes toward the necessity of penal provision for inappropriate handling of genetic information and genetic discrimination (GD) (%)

A													
		2017 (*n* =10,881)					2022 (*n* = 4982)						
		Disagree	Tend to disagree	Cannot say	Tend to agree	Agree	Disagree	Tend to disagree		Cannot say	Tend to agree		Agree
Benefits of using genetic information													
Useful for treatment		3.7	5.0	33.1	43.4	14.8	2.9	3.8		32.4	45.5		15.4
Useful for diagnosis		2.9	3.9	28.7	47.2	17.3	2.0	3.1		25.7	49.6		19.6
Useful for disease prevention		2.7	3.5	28.3	47.6	17.8	2.1	2.4		26.5	49.7		19.2
Leads to a reduction of unnecessary medical expenses		3.0	4.8	35.3	41.1	15.8	2.3	4.2		35.9	41.9		15.8
Leads to the development of science		2.2	4.0	32.0	44.7	17.1	1.6	2.3		29.9	46.5		19.7
Concerns about use of genetic information													
Inappropriate handling in medical institutions		2.5	6.4	45.9	32.3	12.9	2.9	7.6		52.2	26.9		10.4
Inappropriate handling in administrative agencies		2.4	6.3	44.3	31.4	15.6	2.5	7.4		49.1	27.0		13.9
GD in marriage and pregnancy		3.3	8.7	47.0	32.2	8.8	3.3	8.4		52.7	28.0		7.6
GD in employment		3.7	10.4	48.4	29.2	8.4	4.0	10.2		51.9	26.4		7.4
GD in insurance		2.8	8.1	45.3	33.3	10.5	3.2	7.7		48.8	30.8		9.5

B													
									2017 (*n* = 10,881)			2022 (*n* = 4982)	
(1)	Providing or reselling genetic information to a third party without permission								57.1			62.5	
(2)	Toughening penalties for leaking genetic information by a doctor or public official without justifiable reason								53.2			56.8	
(3)	Using materials that contain genetic information without permission								49.7			55.2	
(4)	GD in employment								46.8			51.3	
(5)	Obtaining materials that contain genetic information without permission								46.7			53.4	
(6)	Identifying an individual according to genetic information without permission								46.5			54.4	
(7)	GD in insurance								39.2			43.9	
(8)	Others								0.7			0.7	
	Selecting one or more between (1) and (8)								70.7			74.7	
	Not especially necessary								29.3			25.4	

The respondents were required to answer multiple-choice questions

In both surveys, most of the respondents recognized the need for laws with penalties for inappropriate handling of genetic information and GD (Table 2B) and identified the need for enforcement of penalties for the following activities: to prevent the provision or reselling of genetic information to a third party without permission (57.1% and 62.5% in 2017 and 2022, respectively), to prevent GD at work (46.8% and 51.3% in 2017 and 2022, respectively), and to prevent GD in insurance (39.2% and 43.9% in 2017 and 2022, respectively).

Table 3 shows the results of the logistic regression analysis exploring the relationship between the respondents’ characteristics and the need for penalties. Apart from marital status and educational background, the results in both surveys were almost identical. Some respondents had more awareness of the need for penalties, namely those who were female, who were older, who had high subjective or objective knowledge of genetic terms, who perceived more benefits from the use of genetic information and had concerns about the use of genetic information and GD, and who were willing to undergo genetic testing at medical institutions.

**Table 3:** Multivariate logistic regression models displaying variables associated with the need for penalties for inappropriate handling of genetic information and genetic discrimination (GD) (%)

		2017 (*n* = 10,881)		2022 (*n* = 4982)	
		OR	95% CI		95% CI
Sex	Male	Ref.		Ref.	
	Female	1.50	1.36–1.64	1.64	1.42–1.89
Age group (years)					
20–29		Ref.		Ref.	
30–39		1.19	1.02–1.39	1.31	1.04–1.65
40–49		1.34	1.15–1.56	1.94	1.54–2.45
50–59		1.76	1.49–2.08	2.11	1.66–2.68
60–69		2.39	2.02–2.83	3.32	2.55–4.32
Marital status					
Unmarried		Ref.		Ref.	
Married		0.83	0.73–0.95	0.94	0.76–1.15
Do you have any children?					
No		Ref.		Ref.	
Yes		1.07	0.95–1.20	1.08	0.89–1.31
Educational background					
Junior high school or high school		Ref.		Ref.	
Occupational school or junior college		0.90	0.80–1.01	1.12	0.94–1.33
University or graduate school		0.90	0.81–1.00	1.23	1.04–1.45
Subjective knowledge of the term “genetic”^*1,*3^					
Low or medium		Ref.		Ref.	
High		2.11	1.87–2.39	1.79	1.49–2.15
Objective knowledge of the term “genetic”^*2,*3^					
Low or medium		Ref.		Ref.	
High		1.72	1.52–1.94	2.04	1.70–2.45
Perceived benefits from the use of genetic information^*3^					
Low or medium		Ref.		Ref.	
High		2.88	2.50–3.32	2.73	2.21–3.38
Perceived concerns about the use of genetic information^*3^					
Low or medium		Ref.		Ref.	
High		2.43	2.18–2.71	2.08	1.74–2.49
Willingness to undergo genetic testing at medical institutions					
No or “I cannot decide”		Ref.		Ref.	
Yes		1.65	1.46–1.85	1.78	1.44–2.19

^*1^Subjective knowledge was coded as 2 for “know the meaning of the term,” 1 for “aware of the term,” and 0 for “never heard of the term,” with a score range of 0–10

^*2^Objective knowledge was coded as 1 for a correct answer and 0 for an incorrect answer or “don’t know,” with a score range of 0–5

^*3^The respondents were divided into two groups by the 75th percentile of the score

Our study revealed that in the period between the surveys, the Japanese public’s expectations of genomic medicine and desire for legal regulations, including penalties, had increased; however, their concerns about the use of genetic information and GD had decreased. A survey about attitudes toward the whole genome sequence conducted in 2021 showed that cancer patients and their families had more concerns about the use of genetic information and GD than the general public [17]. Given that the extent of concern about the use of genetic information and GD among respondents with health conditions or a family history of disease, or both was unclear, it is necessary to exercise caution when interpreting our result that public concerns about the use of genetic information and GD have decreased.

Our findings show that female and older respondents, those with higher genetic knowledge, and those with a higher degree of recognition of the benefits of using genetic information and concerns about GD were more likely to express the need for penalties. In previous research, it was found that female and older people had more privacy concerns in North America [18]. Female and older people may strongly feel the need for penalties for their privacy concerns. We also found that highly literate people and those who wanted to receive genomic medicine sought new laws. About 40% of the respondents called for laws and regulations covering GD in insurance, which was lower than that for other activities. Previous research which compared public attitudes towards genetic testing over the years in the Netherlands reported that worries about the insurance companies would demand a genetic test result to calculate health insurance premiums have decreased after the introduction of universal health insurance [19]. The sense of urgency about private insurance may not have been as strong among the respondents because of Japan’s universal health insurance system. Although around half of our respondents called for regulations to cover employment, no discussions have taken place between the government and industrial physicians, employers, or labor unions. Therefore, deliberations on the distinction between the beneficial and discriminatory treatment of employees should be initiated as soon as possible.

It has been pointed out that more serious concerns exist with respect to GD in the context of marriage and pregnancy in Asia than in North America and Europe [4]. The survey results showed that about 40% of the respondents had concerns about GD in marriage and pregnancy, and these cannot be ignored, especially in Asia. Enshrining the refusal of political authorities to tolerate any GD in law may stimulate education about and awareness regarding respect for the human genome and its diversity and contribute to reducing private concerns.

## The need for public engagement against GD

Our surveys had some limitations. The respondents’ experiences of GD were based on self-recognition, which is difficult to verify objectively. Our study was also limited by a low response rate. This may indicate a lack of interest in genetic research and genetic testing. Nonetheless, our surveys were the first to examine public attitudes toward legal regulations regarding GD in Japan.

In Japan, a principled bill will constitute a starting point from which to discuss definitions of GD and examples of GD that should be eliminated from society. As noted by Joly et al. (2022), creating a law prohibiting GD does not necessarily prevent all instances of GD [20]. However, in Japan, where anti-discrimination laws are poor, the bill will bring attention to suspected cases of GD, help prevent them, and contribute to social inclusion. It will be essential to engage the public so that they do not have to endure GD. The fact that many of the respondents in our surveys expressed a desire for legislation on GD prohibition with penalties and that their numbers are increasing should be taken seriously. Although the current bill does not include penalties, it may be necessary to include penalties in the course of scrutinizing the collected cases. For example, people’s concerns could be alleviated by amending the Personal Information Protection Law to punish discriminatory use of personal information, and by strengthening penalties for physicians and public officials who breach personal genetic privacy.

The government is responsible for breaking the chains of GD, which can span generations. It is expected that the future definitions and monitoring of unfair and discriminatory treatment will become more complex due to genome editing interventions on germ cells and embryos and advances in behavioral genetics and social science genomics. The government must not be content with merely enacting the current bill; instead, it must constantly strive to collect GD cases, review definitions, and raise awareness.
