# Correction: Is legislation to prevent genetic discrimination necessary in Japan? An overview of the current policies and public attitudes

**DOI:** 10.1038/s10038-023-01175-9

**Published:** 2023-06-19

**Authors:** Kaori Muto, Akiko Nagai, Izen Ri, Kyoko Takashima, Sachie Yoshida

**Affiliations:** 1 Department of Public Policy, The Institute of Medical Science, The University of Tokyo, Minato-ku, Tokyo Japan; 2 Bioethics Section, Center for Clinical Sciences, National Center for Global Health and Medicine, Shinjuku-ku, Tokyo, Japan; 3 Department of Biomedical Ethics, Kyoto Prefectural University of Medicine, Kyoto City, Kyoto Japan

**Keywords:** Ethics, Personalized medicine

Correction to: *Journal of Human Genetics* 10.1038/s10038-023-01163-z, published online 08 June 2023

In the sentence beginning ‘Influenced by UNESCO’s Declaration in 1997’ in this article, the text ‘also the year in which’ should have read ‘one year after’.

The full sentence is as follows.

Influenced by UNESCO’s Declaration in 1997—which was one year after the Eugenic Protection Law of Japan was finally repealed—the Japanese government took a tough stance against GD by releasing the Basic Principles on Human Genome Research in 2000.

Moreover, in the “Acknowledgements” section, the grant number relating to AMED was incorrectly given as JP20bm0904002 and should have been JP22tm0424701.

The original article has been corrected.

